# Impact of disease progression on individual IPSS trajectories and consequences of immediate versus delayed start of treatment in patients with moderate or severe LUTS associated with BPH

**DOI:** 10.1007/s00345-019-02783-x

**Published:** 2019-05-11

**Authors:** Salvatore D’Agate, Timothy Wilson, Burkay Adalig, Michael Manyak, Juan Manuel Palacios-Moreno, Chandrashekhar Chavan, Matthias Oelke, Claus Roehrborn, Oscar Della Pasqua

**Affiliations:** 1 Clinical Pharmacology and Therapeutics Group, University College London, London, UK; 2 PAREXEL International, Durham, NC USA; 3 Classic and Established Products, GSK, Istanbul, Turkey; 4 Classic and Established Products, GSK, Washington, DC, USA; 5 Classic and Established Products, GSK, Madrid, Spain; 6 Classic and Established Products, GSK, Worli, Mumbai, India; 7 Department of Urology, St. Antonius Hospital, Gronau, Germany; 8 Department of Urology, University of Texas Southwestern Medical Center, Dallas, TX USA; 9 Clinical Pharmacology Modelling and Simulation, GSK, Stockley Park, 1-3 Ironbridge Road, Uxbridge, Middlesex UB11 1BT UK

**Keywords:** Benign prostatic hyperplasia, Clinical trial simulation, Drug–disease modelling, Dutasteride, Lower urinary tract symptoms, Tamsulosin

## Abstract

**Purpose:**

Despite superiority of tamsulosin–dutasteride combination therapy versus monotherapy for lower urinary tract symptoms due to benign prostatic hyperplasia (LUTS/BPH), patients at risk of disease progression are often initiated on α-blockers. This study evaluated the impact of initiating tamsulosin monotherapy prior to switching to tamsulosin–dutasteride combination therapy versus immediate combination therapy using a longitudinal model describing International Prostate Symptom Score (IPSS) trajectories in moderate/severe LUTS/BPH patients at risk of disease progression.

**Methods:**

Clinical trial simulations (CTS) were performed using data from 10,238 patients from Phase III/IV dutasteride trials. The effect of varying disease progression rates was explored by comparing profiles on- and off-treatment. CTS scenarios were investigated, including a reference (immediate combination therapy) and six alternative virtual treatment arms (delayed combination therapy of 1–24 months). Clinical response (≥ 25% IPSS reduction relative to baseline) was analysed using log-rank test. Differences in IPSS relative to baseline at various on-treatment time points were assessed by *t* tests.

**Results:**

Delayed combination therapy initiation led to significant (*p* < 0.01) decreases in clinical response. At month 48, clinical response rate was 79.7% versus 74.1%, 70.3% and 71.0% and IPSS was 6.3 versus 7.6, 8.1 and 8.0 (switchers from tamsulosin monotherapy after 6, 12 and 24 months, respectively) with immediate combination therapy. More patients transitioned from severe/moderate to mild severity scores by month 48.

**Conclusions:**

CTS allows systematic evaluation of immediate versus delayed combination therapy. Immediate response to α-blockers is not predictive of long-term symptom improvement. Observed IPSS differences between immediate and delayed combination therapy (6–24 months) are statistically significant.

**Electronic supplementary material:**

The online version of this article (10.1007/s00345-019-02783-x) contains supplementary material, which is available to authorized users.

## Introduction

The primary treatment goal for lower urinary tract symptoms associated with benign prostatic hyperplasia (LUTS/BPH) is to reduce bothersome symptoms, with clinical management now focused on altering disease progression and preventing long-term complications such as surgery or acute urinary retention (AUR) [1–3]. Treatment guidelines for patients at risk of progression centre on two drug classes: 5α-reductase inhibitors (5-ARI) and α-blockers [4]. These recommendations are supported by data from large clinical studies, which indicate a significant reduction in risk of AUR and BPH-related surgery in patients treated with combination therapy [5, 6]. Additionally, the 4-year Combination of Avodart and Tamsulosin (CombAT) study showed that the dutasteride–tamsulosin combination was more effective than tamsulosin monotherapy in reducing the relative risk of AUR, BPH-related surgery, and BPH clinical progression in men with moderate-to-severe LUTS at increased risk of progression [7].

Following a LUTS/BPH diagnosis, patients are often treated initially with an α-blocker rather than combination therapy, including those at risk of disease progression. Whilst α-blockers may provide immediate symptomatic improvement, it is not possible to predict which patients will show sustained response over time, even if higher baseline symptoms [8] and/or larger prostates [9] are prognostic for deterioration of symptoms. No baseline or clinical factors have yet been identified that are sufficiently sensitive and specific to discriminate progression risk from long-term treatment response in individual patients [10]. Delaying combination therapy may reduce long-term benefits due to the absence of a disease-modifying moiety in the initial treatment phase.

The implications of deviating from clinical guidelines about the use of combination therapy may have been overlooked. Currently, treatment response is mostly assessed in clinical trials and clinical practice by the absolute change from baseline on the International Prostate Symptom Score (IPSS) compared to placebo or active therapy. However, this does not provide insight into the effect of treatment on the rate of progression over time [11, 12]; rather, it describes the magnitude of change over a predefined interval or time span. With respect to the profile of the IPSS measurements over time, any attempt to linearise or interpolate underlying progression rates from changes observed between consecutive visits may lead to inaccurate estimates of disease progression [11].

Recently, we developed a longitudinal model using randomised clinical trial data and follow-up studies including placebo, tamsulosin, dutasteride and combination therapy, which enabled the characterisation of individual IPSS trajectories or profiles [13]. This model provides an opportunity to evaluate the impact of different interventions, taking into account the role of other covariate factors known to affect response in patients with LUTS/BPH with moderate or severe symptoms.

Here, we use computer simulations in conjunction with baseline characteristics from patients participating in previous clinical trials to characterise the deterioration of symptoms associated with varying rates of disease progression and evaluate the long-term effects of different interventions based on individual IPSS trajectories. More specifically, we aim to assess the implications of delayed start of combination therapy in patients with moderate and severe symptoms.

## Patients and methods

Initially, simulations were performed using the longitudinal model recently developed by D’Agate et al. [13] to illustrate the implications for treatment response when drugs with disease-modifying properties are used in patients with varying rates of disease progression. Further details of the model, including a description of the parameters and its implementation for the initial set of simulations are presented in the Supplemental Materials. Based on this preliminary evaluation, baseline IPSS and disease progression rates interact with treatment effect, making it difficult to disentangle the contribution of each factor to the overall response at the end of a predefined interval, as assessed in most clinical trials. These findings support the use of clinical trial simulations (CTS) based on individual IPSS trajectories to assess the deterioration of symptoms associated with varying rates of disease progression and evaluate the effect of early versus delayed onset of combination therapy with the tamsulosin and dutasteride.

**Fig. 1:**
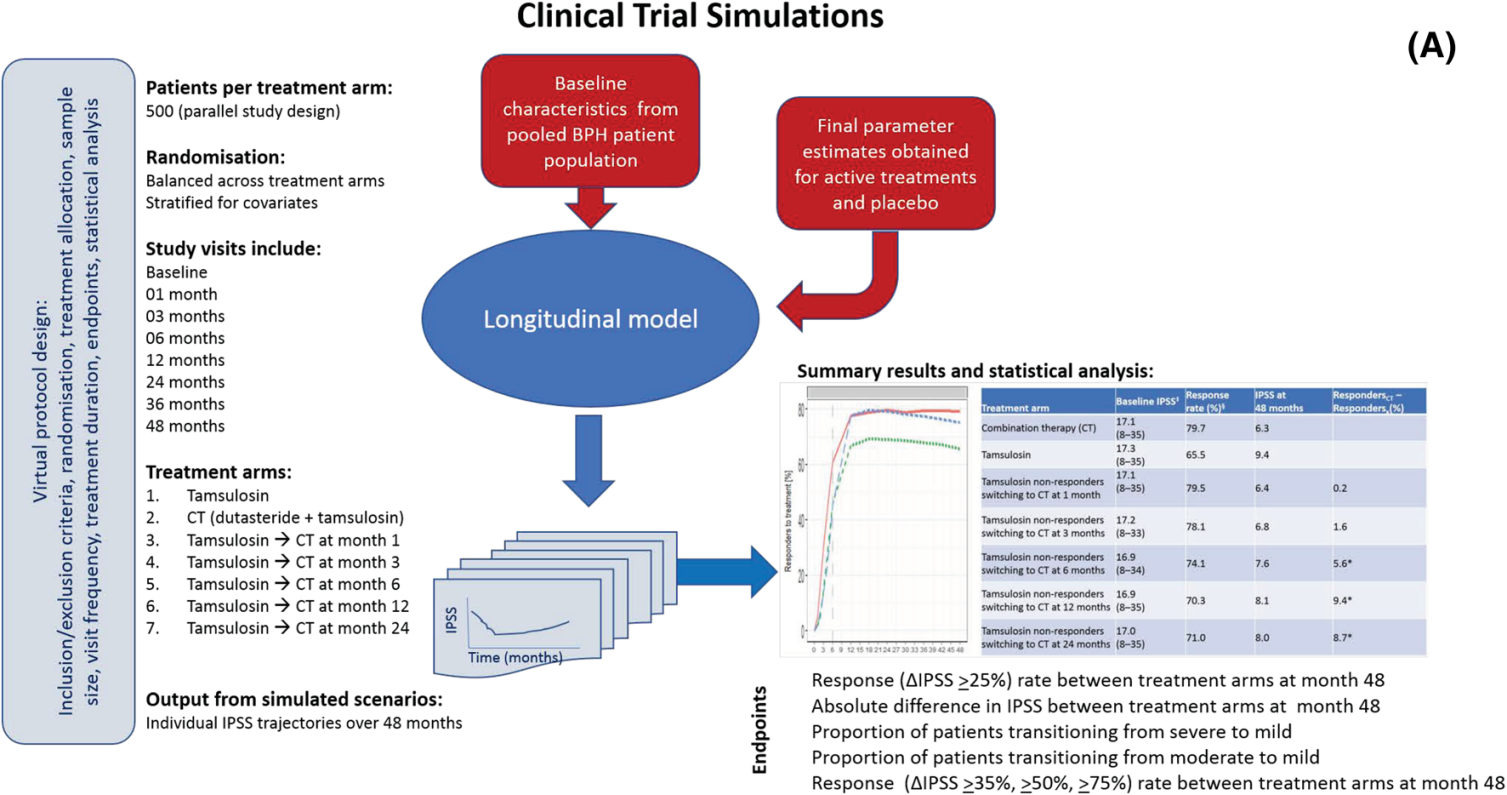

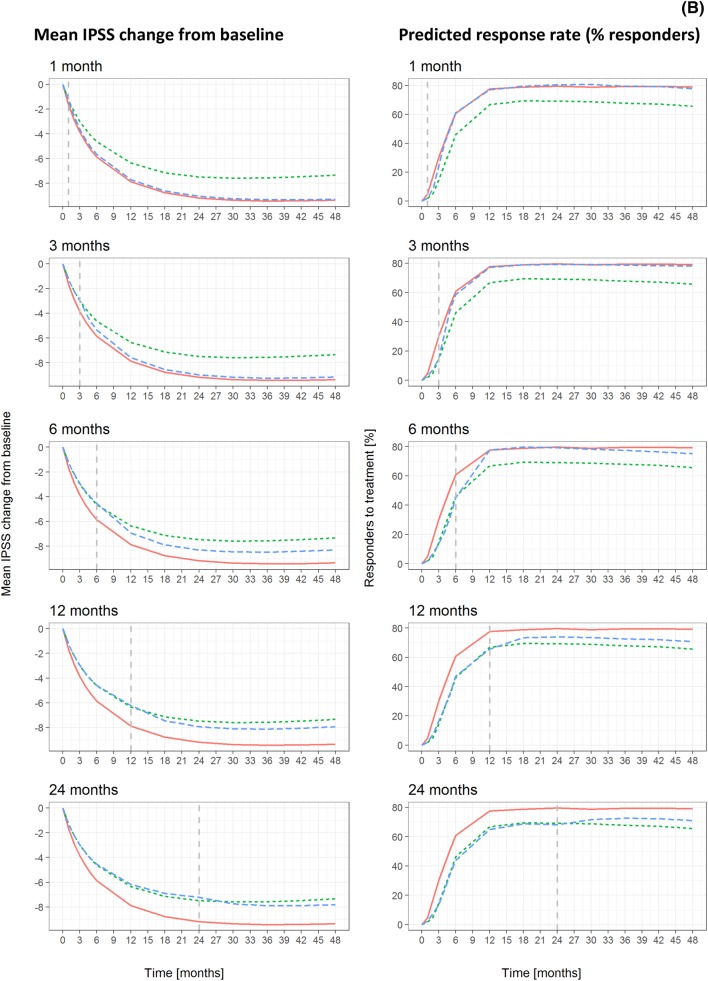

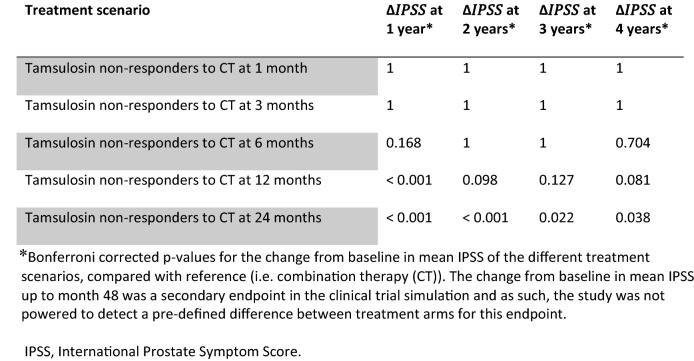
**a** Schematic diagram of CTS based on the longitudinal model describing individual IPSS trajectories [13]. **b** Mean IPSS changes from baseline (left panels) and predicted response rate (% responders) (right panels) stratified by treatment scenario according to a parallel-study design. From top to bottom, each panel depicts the predicted profiles for tamsulosin monotherapy (green dotted line), tamsulosin–dutasteride combination therapy (red solid line), and treatment arm switching to combination therapy (blue dashed line) at 1, 3, 6, 12 and 24 months. The lines depicting tamsulosin monotherapy and tamsulosin–dutasteride combination therapy are constant and represent these treatments being applied throughout 48 months. The dashed vertical lines indicate time of switch to combination therapy. Graphical summaries and statistical analysis refer to the results of a single replicate trial. In these simulations, the placebo effect was assumed to occur only immediately after enrolment into the study. Placebo effect is a key component of the initial response and can last longer than 6 months, as assessed by its half-life. No studies included a placebo treatment arm for > 2 years, so it was not possible to establish whether inter-individual differences might allow for a longer placebo effect

Different CTS scenarios were evaluated, taking into account demographic and clinical covariates known to affect response. The selected scenarios provide insight into the magnitude of treatment response differences following the initiation of combination therapy at different time points. An overview of the CTS steps, including the main protocol design characteristics, data input and output, is presented in Fig. 1a.

### Data source

Demographic and clinical characteristics of the patient pool (*N* = 10,238) included in the CTS were obtained from six Phase III/IV clinical trials (ARIA3001, ARIA3002, ARI40002, CombAT, CONDUCT, ARIB3003). An overview of the patient population is shown in Supplementary Table S1. In CONDUCT, tamsulosin and dutasteride were administered as a fixed-dose combination, while other studies administered treatment as separate dosage forms.

### CTS scenarios

These evaluated the impact of immediate versus delayed start of combination therapy, and focused on the assessment of treatment response defined as the proportion of subjects with a drop in IPSS ≥ 25% from baseline (i.e., the same endpoint in CombAT [14] and CONDUCT [15] studies included in this analysis) to signify a clinically meaningful improvement (CMI) at months 12, 24, 36 and 48. Additionally, the relative difference in the proportion of patients who showed a CMI and transitioned between severity categories was evaluated at months 1, 3, 6, 12, 24 and 48, as described below:

1. Severely symptomatic (IPSS 20–35) to mildly symptomatic (IPSS 1–7).
2. Moderately symptomatic (IPSS 8–19) to mildly symptomatic (IPSS 1–7).

All CTS were performed assuming a protocol design aimed at mimicking clinical guidelines, in which non-responders to α-blockers (patients who had a change in IPSS < 25% of baseline, i.e. who did not experience a CMI) switched to combination therapy at the selected time points. Given the large population sample size included in each scenario, ten replicate trials were considered sufficient to calculate the 90% confidence intervals (CIs) of the estimates. For clarity, graphical summaries were created using a single replicate trial. Full details of the protocol design characteristics are shown in Table S2.

## Results

### Effect of immediate versus delayed start of combination therapy

Demographic characteristics of patients enrolled into each virtual treatment arm are summarised in Table S3. Figure 1b shows the mean IPSS change from baseline and predicted response rate for each treatment scenario. The different curves indicate that switching from tamsulosin monotherapy to combination therapy ≥ 6 months from the start of treatment has a statistically significant effect on the proportion of responders at month 48.

The CTS scenarios confirm the anticipated impact of delaying combination therapy, with the observed differences in IPSS at month 48 appearing to be determined by the delay. The total number of subjects transitioning to combination therapy at the different visits is summarised in Table 1 (panels A and B), along with responder rate at end of the study and 90% CIs from ten trial replicates. From 6 months onwards, a significantly lower proportion of responders was observed in each virtual treatment arm relative to the combination therapy arm. These results are complemented by median estimates of the response rate for a single trial replicate (Table S4). Baseline characteristics of the patients in each treatment arm are summarised to highlight that no other factor appears to predict the lack of response or explain which patients switch from tamsulosin monotherapy after 6, 12 or 24 months.

**Table 1:** CTS results for each treatment arm based on a parallel-study design (ten trial replicates)

(A) Treatment duration	Median number of patients transitioning to combination therapy (90% CI)
Start of treatment	0
01 month	489 (487, 493)
03 months	428 (413, 444)
06 months	284 (266, 306)
12 months	174 (163, 188)
24 months	157 (153, 173)
36 months	0
48 months	0

(B) Treatment arm	Baseline IPSS^a^	Response rate (%)^b^	IPSS at 48 months	Responders_CT_− Responders_*x*_ (%)
Combination therapy (CT)	17.1 [8–35]	79.7 (78.6, 81.2)	6.3 (6, 6.7)	
Tamsulosin	17.3 [8–35]	65.5 (63.1, 68.1)	9.4 (8.8, 9.9)	
Tamsulosin non-responders to CT at 1 month	17.1 [8–35]	79.5 (76.6, 81.4)	6.4 (6.2, 6.6)	0.2
Tamsulosin non-responders to CT at 3 months	17.2 [8–33]	78.1 (76.8, 81.0)	6.8 (6.4, 7.1)	1.6
Tamsulosin non-responders to CT at 6 months	16.9 [8–34]	74.1 (72.3, 75.5)	7.6 (7.2, 8.1)	5.6*
Tamsulosin non-responders to CT at 12 months	16.9 [8–35]	70.3 (67.3, 72.1)	8.1 (7.5, 8.6)	9.4*
Tamsulosin non-responders to CT at 24 months	17.0 [8–35]	71.0 (68.6, 72.1)	8.0 (7.8, 8.3)	8.7*

(C) Treatment arm	Transition from moderate to mild at different study visits, cumulative % (range)					
	1 month	3 months	6 months	12 months	24 months	48 months
Combination therapy (CT)	4.6 (3.8, 5.2)	12.3 (11.2, 13.8)	22.3 (20.5, 23.3)	35.3 (34.4, 37.0)	44.7 (43.4, 46.4)	45.5 (44.2, 46.6)
Tamsulosin	3.0 (2.8, 3.42)	8.0 (6.5, 8.4)	13.6 (13.4, 14.6)	25.2 (23.4, 26.1)	32.9 (32.0, 34.8)	32 (31.0, 34.2)
Tamsulosin non-responders switching to CT at 1 month	4.1 (3.6, 5)	11.0 (10.6, 12.2)	21.6 (20.6, 23.0)	35.1 (34.4, 37.3)	43.3 (41.7, 45.8)	43 (41.4, 46.1)
Tamsulosin non-responders switching to CT at 3 months	3.9 (3.29, 4.4)	7.7 (6.3, 9.2)	17.3 (15.9, 21.0)	33.0 (31.1, 36.1)	41.6 (39.2, 44.6)	40.7 (38.3, 44.0)
Tamsulosin non-responders switching to CT at 6 months	3.2 (2.6, 3.5)	7.6 (7.0, 8.9)	14.4 (12.8, 15.8)	30.5 (29.1, 33.6)	40.7 (38.6, 42.6)	40.4 (37.9, 42.5)
Tamsulosin non-responders switching to CT at 12 months	3.6 (3.4, 4.2)	8.4 (7.4, 9.4)	14.8 (13.2, 15.9)	25.2 (23.0, 27.5)	37.9 (35.9, 40.9)	37.2 (35.7, 40.7)
Tamsulosin non-responders switching to CT at 24 months	3.3 (3.0, 4.2)	8.1 (7.0, 9.6)	14.7 (12.7, 16.4)	25.4 (23.1, 27.0)	34.3 (32.7, 35.0)	38.9 (37.0, 40.6)

(C) Treatment arm	Transition from severe to mild at different study visits, cumulative % (range)					
	1 month	3 months	6 months	12 months	24 months	48 months
Combination therapy (CT)	0 (0, 0.1)	0.4 (0.2, 0.8)	2.2 (1.6, 2.8)	6.6 (4.8, 7.1)	12.8 (11.5, 13.4)	15.7 (13.8, 16.4)
Tamsulosin	0 (0, 0)	0.2 (0, 0.7)	1.1 (0.8, 1.5)	2.8 (2.6, 4.3)	6.8 (6.1, 9.0)	10.2 (9.0, 12.2)
Tamsulosin non-responders switching to CT at 1 month	0 (0, 0)	0.4 (0, 1.0)	1.8 (1.6, 2.9)	6.8 (5.3, 8.0)	13.3 (12.2, 14.8)	16.7 (15.2, 17.2)
Tamsulosin non-responders switching to CT at 3 months	0 (0, 0.4)	0.2 (0, 0.5)	1.3 (1.0, 2.3)	6.3 (5.0, 8.0)	13.6 (11.5, 15.4)	16.2 (14.6, 18.4)
Tamsulosin non-responders switching to CT at 6 months	0 (0, 0)	0.3 (0, 0.7)	0.9 (0.8, 1.9)	4.3 (3.0, 4.9)	9.3 (8.6, 10.7)	11.6 (10.8, 12.9)
Tamsulosin non-responders switching to CT at 12 months	0 (0, 0.2)	0.4 (0.2, 0.6)	1.0 (0.8, 1.2)	3.4 (2.2, 4.4)	9.4 (7.1, 10.1)	12.6 (9.7, 13.1)
Tamsulosin non-responders switching to CT at 24 months	0 (0, 0.1)	0.3 (0.2, 0.6)	1.2 (0.8, 1.7)	3.0 (2.6, 4.0)	7.1 (6.4, 8.2)	10.6 (9.8, 12.0)

(D) IPSS improvement	Percentage of patients (CI) showing improvement at month 48			
	≥ 25%	≥ 35%	≥ 50%	≥ 75%
Combination therapy (CT)	79.7 (78.6, 81.2)	73.1 (71.6, 74.8)	60.8 (59.0, 62.4)	31.2 (27.4, 32.6)
Tamsulosin	65.5 (63.0, 68.0)	57.4 (55.6, 60.0)	44.0 (42.5, 46.5)	17.0 (15.2, 18.6)
Tamsulosin non-responders switching to CT at 1 month	79.5 (76.5, 81.4)	71.1 (69.3, 74.7)	59.3 (56.6, 60.9)	29.1 (27.2, 29.9)
Tamsulosin non-responders switching to CT at 3 months	78.1 (76.8, 81.0)	71.0 (69.9, 73.9)	59.9 (56.3, 61.2)	28.4 (25.8, 31.3)
Tamsulosin non-responders switching to CT at 6 months	74.1 (72.3, 75.5)	65.8 (64.1, 68.6)	52.7 (50.6, 55.6)	22.4 (20.3, 24.2)
Tamsulosin non-responders switching to CT at 12 months	70.3 (67.2, 72.1)	62.4 (58.7, 64.6)	48.4 (45.1, 51.9)	20.2 (18.1, 24.4)
Tamsulosin non-responders switching to CT at 24 months	71.0 (68.5, 72.1)	63.2 (61.3, 65.0)	49.6 (46.9, 52.2)	19.4 (18.0, 21.0)

Panel (A): overview of the patient population transitioning from tamsulosin to tamsulosin–dutasteride combination therapy due to non-response to tamsulosin. Panel (B): primary endpoint, i.e. proportion of responders (response rate) and IPSS values at 48 months in patients responding to treatment. The difference in the proportion of responders in each virtual treatment arm relative to the combination therapy arm [Responders_CT _− Responders_*x*_ (%)] summarises the impact of immediate combination therapy. Panel (C): cumulative percentage of subjects switching from moderate or severe to mild symptom scores at each visit. Panel (D): Impact of immediate versus delayed start of tamsulosin–dutasteride combination therapy on the magnitude of response, as assessed by the proportion of patients showing changes in IPSS ≥ 35%, ≥ 50% and ≥ 75% relative to baseline at month 48. The statistical significance of the differences between treatment arms for each response threshold is shown for a single replicate trial in Table S5 (see Supplemental Materials)

The results presented above refer to a CTS scenario including placebo effect only after the initial treatment phase. Placebo effect is an important component of the initial response and can last more than 6 months, as assessed by its half-life. No studies included a placebo treatment arm for > 2 years, so it was not possible to establish whether inter-individual differences might allow for a longer placebo effect. Unless indicated otherwise, values represent median (90% CIs) from ten trial replicates. Symptom severity: mild = IPSS 1–7, moderate = IPSS 8–19, and severe = IPSS ≥ 20

*CI* confidence interval, *CTS* clinical trial simulation, *IPSS* International Prostate Symptom Score

*Log rank test: *p *< 0.01

^a^Baseline IPSS [range] in each treatment arm

^b^percentage of responders (IPSS drop ≥ 25% relative to baseline) at 48 months

As summarised in Table 1 (panels C and D), the impact of the delayed start of combination treatment is also reflected in the total number of patients who transition from severe or moderate to mild IPSS categories over the 48-month period. In addition, the lower panel in Table 1 provides further evidence of the difference in magnitude of clinical improvement, assessed by percentage of responders per treatment arm with a decrease in IPSS ≥ 25%, ≥ 35%, ≥ 50% or ≥ 75% relative to baseline. These results indicate that a significant proportion of patients showed greater improvement in symptoms when combination therapy was started immediately.

## Discussion

Meta-analyses have been used to compare different treatment options. However, this technique allows scrutiny only of design factors that have been implemented, without necessarily correcting for the effect of confounding factors which cannot be easily excluded. Moreover, they often focus on mean parameter estimates, yielding results that ignore underlying covariates that may modify the treatment effect. By contrast, the application of longitudinal modelling and CTS at individual patient level allows investigation of a range of design characteristics on the power to detect treatment effects, without confounding or practical restrictions, prior to exposing patients to an experimental intervention [16, 17]. CTS can be performed not only to evaluate scenarios that have not been previously investigated in clinical trials, but also to explore hypothetical scenarios which cannot be implemented in real-life conditions. Indeed, the implementation of a prospective, controlled study in which combination therapy is delayed may be ethically questionable, especially when guidelines recommend it in patients considered at risk for progression of BPH [4]. Here we have shown how this methodology can be used to explore design factors, such as delayed start of treatment, whilst disentangling it from other factors and interactions. Our analysis also provided an opportunity to assess the effect of disease progression, baseline covariates, and drug treatment on individual IPSS trajectories.

### Effect of disease progression, baseline covariate factors and drug treatment on individual IPSS trajectories

Notwithstanding the body of evidence regarding the benefits of tamsulosin–dutasteride combination therapy, including greater and more durable improvement than with either monotherapy [14, 18, 19], little attention has been given to the impact of variable rates of disease progression on treatment response or deterioration of symptoms, as measured by IPSS [10, 20, 21]. There are currently no reliable biomarkers that allow identification and prediction of a specific clinical phenotype for disease progression in individual patients, although serum PSA has been explored in this capacity [8, 9]. This is further compounded by limited understanding of the effects of specific comorbidities or other covariates on overall treatment response [22]. Our analysis suggests these limitations may partly be overcome by further characterisation of individual IPSS trajectories.

The introduction of IPSS as a tool for clinical practice and in research protocols was originally based on data from relatively short-term validation steps [23]. Among the available reports on the natural history of LUTS, long-term longitudinal follow-up studies have been restricted to changes in IPSS relative to baseline, making it difficult to distinguish the impact of multiple interacting factors on the deterioration of symptoms. Exploratory investigations have suggested that IPSS does not correlate with laboratory measures of urinary function at baseline (e.g., PV and PSA), but limitations in the methodology employed indicate that this should not be taken as conclusive [24, 25]. In fact, the lack of such correlations has been often assigned to study design, inclusion and exclusion criteria, and sample size. Nevertheless, baseline IPSS has been shown to predict the risk of progression in several prior clinical trials [6–8].

These apparently paradoxical findings may reflect the empirical nature of past research protocols, which assess or infer disease progression directly from the observed increase in IPSS over time, rather than based on a specific parameter that captures the underlying processes [26–28]. In this context, the initial simulations show the implications of the interaction between baseline covariates (e.g. IPSS) and varying rates of disease progression on individual trajectories (see Supplemental Materials). Although the possibility that patients with LUTS/BPH may show intrinsic differences in response to α-blockers and 5ARI cannot be excluded, it is clear from figures S1, S2 and S3 that the identification of inter-individual variation in progression rates only from baseline characteristics may be challenging. Further exploration is required to identify whether baseline markers may explain some of this heterogeneity in symptom deterioration and consequently in treatment response.

### Clinical trial simulations: impact of immediate versus delayed start of combination therapy

While the use of combination therapy in LUTS/BPH patients with moderate and severe symptoms at risk of disease progression is endorsed by international guidelines, this analysis was performed taking into account prior evidence of the favourable benefit–risk profile of dutasteride–tamsulosin combination [29]. An early start of treatment with combination therapy should not alter the overall safety profile. However, the current results show that symptomatic treatment does not stop disease progression, and potentially reduces the benefits associated with the 5-ARI disease-modifying properties, if treatment is delayed for more than 6 months.

To address the key research question of this investigation, we have focused on the results of a parallel-study design, which reflects a typical clinical trial setting in which patients are randomised to different treatment arms, even though it does not fully capture the implications of delayed start of combination therapy for every single patient. In this regard, our results do not exclude the contribution of between-patient variability on predicted treatment response. It is worth mentioning that baseline characteristics among tamsulosin non-responders do not differ significantly from patients on combination therapy raising questions as to whether baseline demographics and clinical features may be sufficiently discriminative in determining treatment response. Yet, early administration of tamsulosin–dutasteride combination therapy (i.e. < 6-month delay) not only results in a significantly greater responder rate at month 48 in men at risk of progression, but also leads to an increase in the fraction of patients transitioning to lower IPSS severity levels. More strikingly, early use of combination therapy results in a larger proportion of patients having greater clinical improvement, i.e. changes in IPSS relative to baseline (Table 1, panels C and D). Early start of combination treatment allows ~ 10% more patients to benefit from symptomatic improvement.

The clinical relevance of differences in IPSS may be questioned without careful understanding of the approach used here. First, the definition of response based on a ≥ 25% reduction in IPSS relative to baseline allows for a standardised measure across the entire population, irrespective of symptom severity at baseline, provided that the response strongly influences the relationship between the perception of improvement and the score improvement. This threshold of ≥ 25% reduction in IPSS has been used as a study endpoint in various clinical trials included in this CTS [14, 15]. Second, CTS were performed without residual noise to ensure accurate estimates of the effect of the different interventions. Residual noise reduces the sensitivity to detect a true treatment response.

Even though a few assumptions are required to assess treatment response when designing a standard clinical trial protocol or a virtual one, as in our case, our results highlight that immediate exposure to tamsulosin–dutasteride combination therapy can have a long-lasting impact on the individual trajectories of a small, but clinically relevant fraction of patients (i.e. those who have a faster disease progression rate but cannot be identified at start of treatment). Considering the chronic nature of the disease, the effect of disease-modifying properties cannot be compensated by symptomatic interventions over the longer term.

Undeniably, there are limitations in the work performed. First, it should be recognised that the population used for the development of the longitudinal model represents a subgroup of the overall BPH patient population, and as such encompasses only subjects with moderate or severe symptoms at risk of progression. In addition, we have assumed that the placebo effect starts at the beginning of the trial and not when the therapy is switched at later time points. As the longitudinal model used for the simulations showed some bias in terms of the predicted trajectories severe patients with very high IPSS scores (i.e., upper 2.5th percentile), there is a chance of misclassification of non-responders. Last, we have not evaluated IPSS profiles beyond 48 months to ensure that simulation results could be supported by existing clinical data, i.e., the time span used in the analysis matches the duration of the longest clinical trial included in the development of the model.

## Conclusions

We demonstrated how CTS can be used to describe individual IPSS trajectories and explore the implications of immediate versus delayed start of tamsulosin–dutasteride combination therapy in men at risk of disease progression. Importantly, simulation scenarios showed higher overall response rates and significantly lower IPSS values following immediate combination therapy. Initiation of monotherapy in LUTS/BPH patients with moderate or severe symptoms, who are at risk of disease progression, may offer less advantage; it appears to reduce the long-term benefit from early combination therapy. Patients eligible to initiate combination therapy may miss the symptomatic benefit over the long term, as shown by the significant difference in proportion of responders was observed in the treatment arms, when switching from tamsulosin occurs at 6 months or later.

## Electronic supplementary material

Below is the link to the electronic supplementary material.

Supplementary material 1 (PDF 1274 kb)
