# Supplementary material for: Impact of disease progression on individual IPSS trajectories and consequences of immediate versus delayed start of treatment in patients with moderate or severe LUTS associated with BPH
Source: World J Urol. 2019 May 11;38(2):463–72. doi: 10.1007/s00345-019-02783-x (PMC6994451; doi:10.1007/s00345-019-02783-x)
Supplement: Supplementary file 1 — Supplementary material 1 (PDF 1274 kb) [file 345_2019_2783_MOESM1_ESM.pdf]

Supplementary Material

Prior to the implementation of clinical trial simulations, we have attempted to evaluate the advantages of a parametric approach, integrating disease progression, placebo effect and treatment effect to describe individual IPSS trajectories. Because model parameterisation is based on distinct terms for disease progression and treatment effect, it was possible to assess how different rates of progression induce the deterioration of symptoms and affect overall treatment response, and to distinguish symptomatic improvement in IPSS from the disease-modifying effects of treatment.

Longitudinal model: The longitudinal model describing the individual trajectory and progression of IPSS was developed using turnover concepts with a Gompertz model (Equation 1) [1]. Treatment effect was then parameterized relative to the placebo intervention as covariate effects on disease progression rate (Equation 2-3).

$$\frac{d(IPSS)}{dt} = \left(DISP \cdot \left(1 - \frac{IPSS}{35}\right)\right) - \left((PLACEBO + TREATMENT) \cdot IPSS\right), IPSS(0) = IPSS_b \quad \textbf{(Equation 1)}$$

$$PLACEBO = DELTA_{placebo} \cdot e^{-TIME \cdot \frac{\ln(2)}{T_{\frac{1}{2}}}} \quad \textbf{(Equation 2)}$$

$$TREATMENT = DELTA_{treatment} \quad \textbf{(Equation 3)}$$

The term dIPSS/dt in Equations 1 represents the rate of change (ie, derivative) in IPSS. *DISP* represents the coefficient describing the rate of progression or degeneration of symptoms (ie, disease progression rate). The $DELTA_{placebo}$ term represents the maximum rate of reduction of symptoms due to placebo intervention and $T_{1/2}$ is the half-life of the effect of the placebo intervention. The term $DELTA_{treatment}$ accounts for the effect of any active intervention in an additive manner to the underlying disease progression rate and placebo effect. $IPSS_0$ is the observed IPSS at baseline, $IPSS_b$ is the baseline disease state. This model parameterization provides a more physiological interpretation of the effect of the intervention on disease progression (i.e., on the individual IPSS trajectory).

Full details of the model-building and evaluation have been described elsewhere [2]. It should be noted that for the purposes of the current analysis, simulations were performed using parameter estimates from a final model in which the observed IPSS at baseline ($IPSS_0$) were assumed to reflect baseline disease state ($IPSS_b$). Alternative procedures can also be applied to estimate $IPSS_b$ if one assumes that the underlying disease state is unknown at the initiation of treatment [3]. Both approaches yield comparable model parameters estimates describing the individual IPSS trajectories.

Simulation-based assessment of the effect of disease progression and treatment on individual IPSS trajectories

Initially, simulations were performed to illustrate implications for treatment response when drugs with disease-modifying properties are used in individual patients with varying disease-progression rates along with the predicted profiles in the absence of any active treatment (Supplementary Figure S1). Because combination therapy produces both symptomatic and disease-modifying effects, the resulting treatment response will depend on the underlying progression rate. In fact, different percentiles of the disease

progression (*DISP)* parameter distribution were used to visualise and distinguish between the range of possible IPSS values due to the underlying progression of disease and treatment response. The interaction between these two factors can be further characterised by the net change from baseline (ΔIPSS) over time. In addition, to assess the impact of disease state, as defined by IPSS severity at baseline, the effect of symptomatic and disease-modifying effects of tamsulosin monotherapy (i.e. symptomatic treatment) and tamsulosin-dutasteride combination therapy (i.e. symptomatic + disease-modifying treatment) on individual IPSS trajectories was simulated and stratified by baseline IPSS (Supplementary Figures S2 and S3). These profiles demonstrate that baseline IPSS and disease-progression rates interact with treatment effect, making it difficult to disentangle the contribution of each factor to response, which in a typical clinical trial is often defined in terms of relative change from baseline.

It should be noted that in a typical clinical trial setting, appraisal of the rate of progression and its implication for treatment response is confounded by residual variability in IPSS. The relevance of model-predicted individual IPSS trajectories is emphasised in Supplementary Figure S4, where the impact of measurement noise on the evaluation of IPSS after administration of combination therapy is illustrated for patients with mild, moderate or severe symptoms.

These results also explain why no predefined set of baseline characteristics has been identified as a sufficiently sensitive marker of the deterioration of symptoms or treatment response. In fact, in a recent data-mining exercise including men with LUTS secondary to BPH, it was shown that baseline IPSS severity achieved sensitivity and specificity of 70% and ~50%, respectively, as predictors of individual response to placebo or tadalafil [4]. However, these values are below the sensitivity and specificity threshold of 80% that enables reliable allocation of an individual patient to either the responder or non-responder group [4]. Hence, clinicians cannot accurately predict whether a patient will respond to symptomatic and disease-modifying interventions at the start of treatment.

Figure S1. Impact of the disease-modifying properties of tamsulosin-dutasteride combination therapy on the IPSS response in individual patients with varying rates of disease progression. Each panel depicts the IPSS trajectories (upper panels) and the ΔIPSS (lower panels) over 48 months for patients across a range of disease progression rates (2.5th, 25th, 50th, 75th and 97.5th percentiles). Red areas demonstrate predicted profiles in the absence of any active treatment; blue areas demonstrate varying progression rates receiving combination therapy; solid lines are mean predicted IPSS; and shaded areas represent the 95% prediction interval (n=200 simulations). The predicted trajectories describing disease progression are depicted, assuming a hypothetical scenario in which patients remain untreated despite deterioration of symptoms. CTS show predicted IPSS without residual errors.

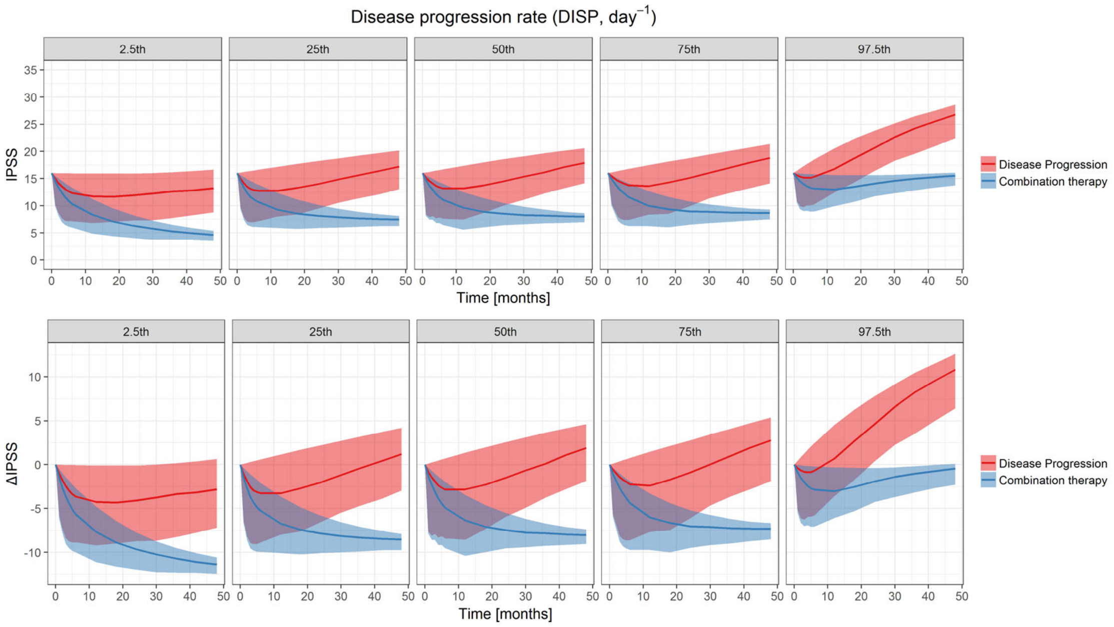

Figure S2. Impact of baseline symptom severity on individual IPSS trajectories, and disease-modifying properties of tamsulosin-dutasteride combination therapy in individual patients with comparable rates of disease progression. IPSS trajectories (upper panels) and ΔIPSS (lower panels) over 48 months are depicted for patients with different baseline IPSS (8, 12, 16, 20 and 30). Red areas demonstrate predicted profiles in the absence of any active treatment; blue areas demonstrate varying progression rates receiving combination therapy; solid lines are mean predicted IPSS; and shaded areas represent 95% prediction intervals (n=200 simulations). The predicted trajectories describing disease progression are depicted, assuming a hypothetical scenario in which patients remain untreated despite deterioration of symptoms. CTS show predicted IPSS without residual errors.

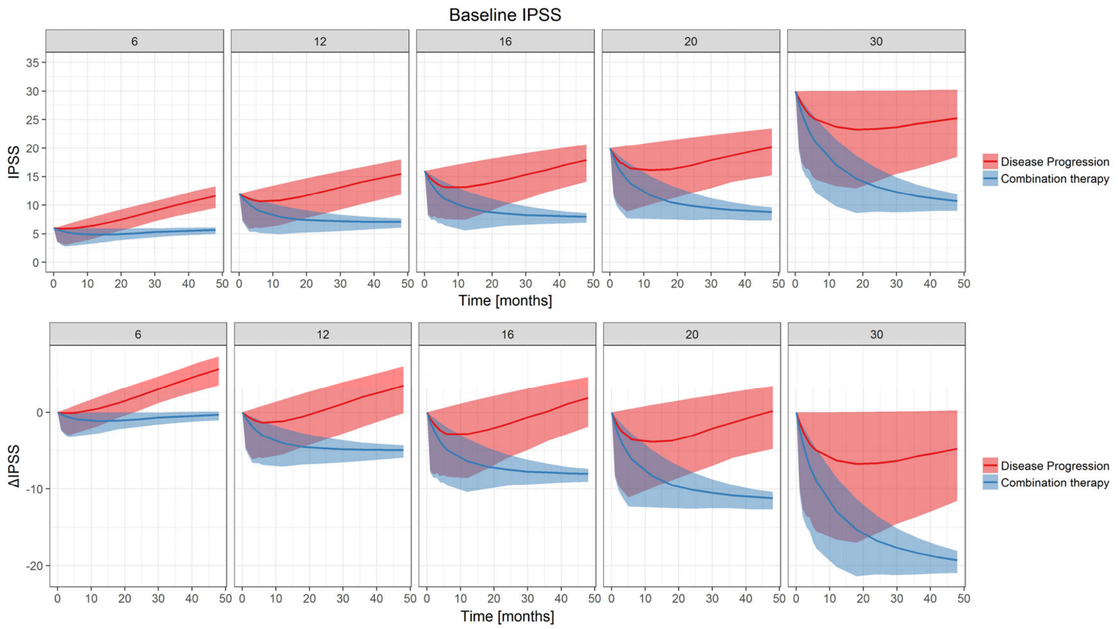

Figure S3. Impact of symptomatic (tamsulosin monotherapy, upper panel) and symptomatic and disease-modifying properties (tamsulosin-dutasteride combination therapy, lower panel) on individual IPSS trajectories in patients with varying rates of disease progression and varying IPSS at baseline. Panels are stratified by symptom severity, as defined by IPSS values at baseline. Red areas demonstrate predicted profiles in the absence of any active treatment; blue areas demonstrate varying progression rates receiving either tamsulosin monotherapy (upper panel) or combination therapy (lower panel); solid lines are mean predicted IPSS; and shaded areas represent 95% prediction intervals (n=200 simulations). The predicted trajectories describing disease progression (red) are depicted, assuming a hypothetical scenario in which patients remain untreated despite deterioration of symptoms.

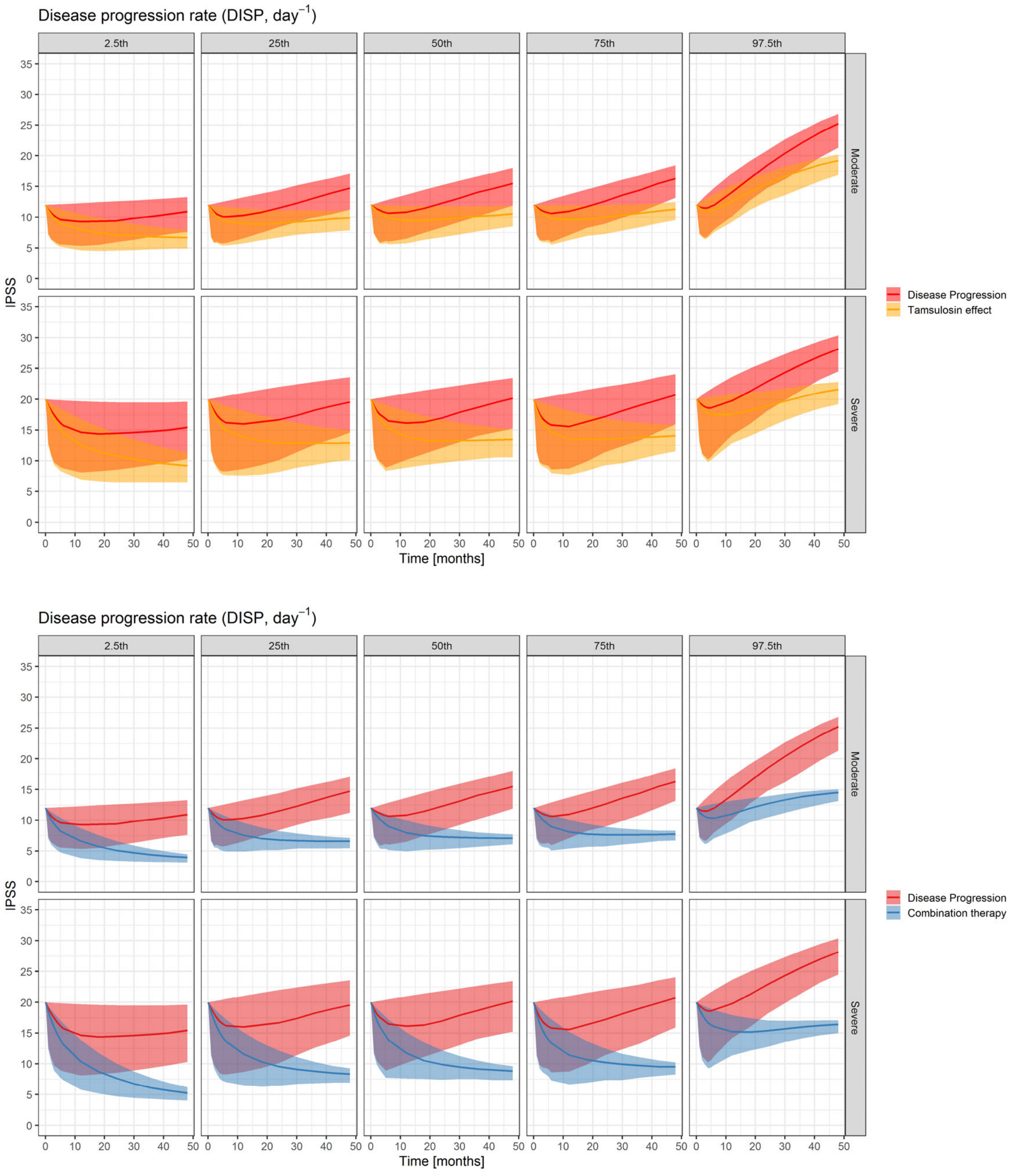

Figure S4. Example of simulated individual IPSS trajectories in patients with varying rates of disease progression, after incorporation of residual variability. Panels are stratified by symptom severity, as defined by IPSS values at baseline. Each panel depicts the predicted (line) and 'observed' (dots) IPSS trajectories over 48 months after administration of tamsulosin-dutasteride combination therapy. The predicted trajectories describing disease progression are depicted, assuming a hypothetical scenario in which patients remain untreated despite deterioration of symptoms.

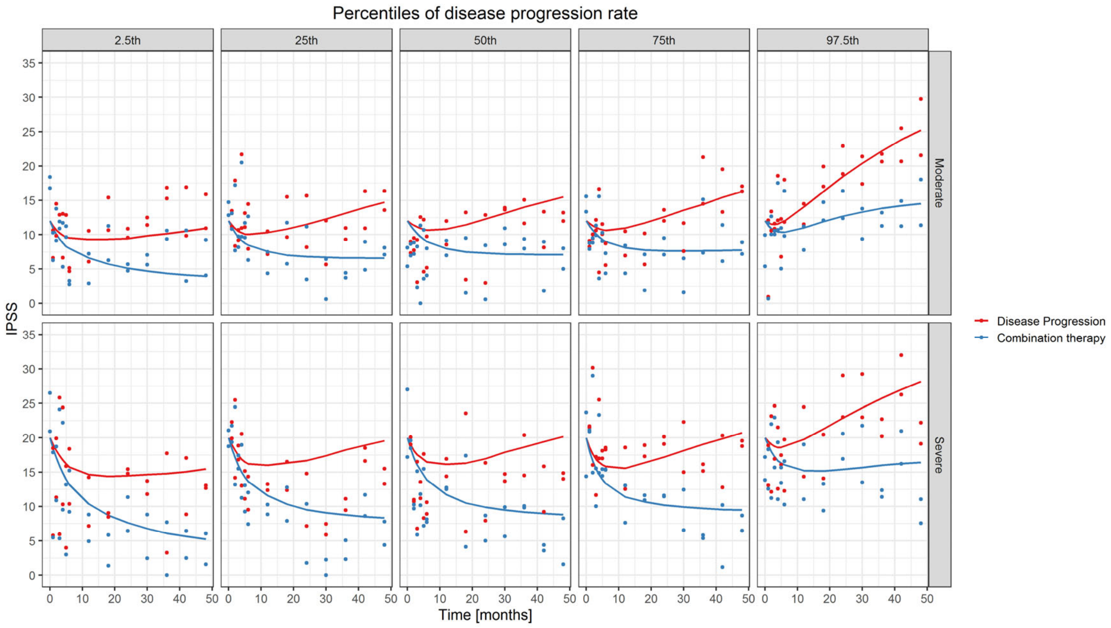

References

[1] Jacqmin P, McFadyen L, Wade JR. Basic PK/PD principles of drug effects in circular/proliferative systems for disease modelling. J Pharmacokinet Pharmacodyn. 2010;37:157-77.

[2] D'Agate S, Wilson T, Chavan C, Adalig B, Manyak M, Palacios-Moreno JM, et al. Abstract 8794 - Development of a drug-disease model describing individual IPSS trajectories in BPH patients: Implication of disease progression and covariate factors on long term treatment response. Annual Meeting of the Population Approach Group in Europe (PAGE) 27; Montreux, Switzerland. 2018.

[3] Holford NH, Chan PL, Nutt JG, Kieburtz K, Shoulson I. Parkinson Study Group. Disease progression and pharmacodynamics in Parkinson disease - evidence for functional protection with levodopa and other treatments. J Pharmacokinet Pharmacodyn. 2006; 33:281-311

[4] Fusco F, D'Anzeo G, Henneges C, Rossi A, Buttner H, Nickel JC. Predictors of Individual Response to Placebo or Tadalafil 5mg among Men with Lower Urinary Tract Symptoms Secondary to Benign Prostatic Hyperplasia: An Integrated Clinical Data Mining Analysis. PLoS One. 2015;10:e0135484.

Table S1. Overview of the studies identified for the proposed model-based meta-analysis. Protocol title is shown, along with details of treatment type and duration, and the purpose of the study data during model-building and validation procedures.

Study number	Study description	Dose and administration	Number of patients	Purpose in this study	Visits (months or weeks)
ARIA3001	A multicentre, randomised, double-blind, placebo-controlled, two-year, parallel-group study with a two-year, open-label phase	o.d. 0.5 mg dutasteride for two years	720	Model building	AUA-SI/IPSS: 0, 1, 3, 6, 12, 18, 24, 30, 36, 42 and 48<br>Prostate volume: 0, 1, 6, 12, 24 and 48<br>Max. urinary flow: 0, 1, 3, 6, 12, 18, 24, 30, 36, 42 and 48<br>PSA: 0, 1, 3, 6, 12, 18, 24, 36 and 48
		o.d. placebo for two years	720	Model building	
ARIA3002	A multicentre, randomised, double-blind, placebo-controlled, two-year, parallel-group study with a two-year, open-label phase	o.d. 0.5 mg dutasteride for two years	677	Model building	AUA-SI/IPSS: 0, 1, 3, 6, 12, 18, 24, 30, 36, 42 and 48<br>Prostate volume: 0, 3, 6, 12, 24, 36 and 48<br>Max. urinary flow: 0, 1, 3, 6, 12, 18, 24, 30, 36, 42 and 48<br>PSA: 0, 1, 3, 6, 12, 18, 24, 36 and 48
		o.d. placebo for two years	685	Model building	
ARI40002	A pilot, multicentre, double-blind, parallel-group, 36-week, randomised study	o.d. 0.5 mg dutasteride & 0.4 mg tamsulosin for 36 weeks	164	Model building	AUA-SI/IPSS: 0, 4, 12, 24, 30, 36 and 37 weeks<br>PSA: 0 and 36 weeks
		o.d. 0.5 mg dutasteride for 12 weeks after 24 weeks. o.d. combination therapy	163	Model building	
CombAT	A multicentre, randomised, double-blind, four-year, parallel-group study	o.d. 0.4 mg tamsulosin for four years	1611	Model building	AUA-SI/IPSS: 0, 3, 6, 9, 12, 15, 18, 21, 24, 27, 30, 33, 36, 39, 42, 45 and 48<br>Prostate volume: 0, 12, 24, 36 and 48<br>Max. urinary flow: 0, 6, 12, 18, 24, 30, 36, 42 and 48<br>PSA: 0, 12, 24, 36 and 48
		o.d. 0.5 mg dutasteride for four years	1623	Model building	

		o.d. 0.5 mg dutasteride & 0.4 mg tamsulosin therapy for four years	1610	Model building	
CONDUCT	A multicentre, randomised, open-label, two–year, parallel-group study	watchful waiting with protocol-defined initiation of o.d.0.4 mg tamsulosin	373	Model building	AUA-SI/IPSS: 0, 1, 3, 6, 9, 12, 15, 18, 21 and 24<br>PSA: 0, 6, 12 and 24
		o.d. 0.5 mg dutasteride & 0.4 mg tamsulosin therapy for two years	369	Model validation	
ARIB3003	A multicentre, randomised, double-blind, placebo-controlled, two-year, parallel-group study with a two-year, open-label phase	o.d. 0.5 mg dutasteride for two years	770	Model validation	AUA-SI/IPSS: 0, 1, 3, 6, 12, 18, 24, 30, 36, 42 and 48<br>Prostate volume: 0, 6, 12, 18, 24 and 48<br>Max, urinary flow: 0, 1, 3, 6, 12, 18 24, 30, 36, 42 and 48<br>PSA: 0, 1, 3, 6, 12, 18, 24, 36 and 48
		o.d. placebo for two years	753	Model validation	

AUA-SI, American Urological Association Symptom Index; IPSS, International Prostate Symptom Score; o.d., once-daily; PSA, prostate-specific antigen

Table S2. Protocol design characteristics used for the simulation of individual IPSS trajectories after immediate and delayed start of treatment with tamsulosin-dutasteride combination therapy:

**Protocol characteristics**	
**Endpoints:**	Individual IPSS trajectories were simulated over 48 months. Endpoints evaluated were: 1) predicted absolute change in IPSS at predefined visits; 2) predicted percentage of subjects with a drop in IPSS ≥25% from baseline i.e. 'responders'; those patients who experienced a CMI [29]; and 3) predicted percentage of patients transitioning from severe or moderate to mild IPSS severity.
**Patient baseline characteristics:**	The patient population randomised and assigned to each CTS was generated taking into account similar inclusion/exclusion criteria as used for the main combination-treatment studies, CombAT and CONDUCT [15, 20]. Inclusion criteria (at enrolment) were: men aged ≥50 years, IPSS ≥8 points, prostate volume ≥30 cm^3, total serum PSA ≥1.5 ng/mL to <10 ng/mL, maximum urine flow >5 mL/s to ≤15 mL/s with a minimum voided volume ≥125 mL. Exclusion criteria comprised history or evidence of prostate cancer, previous prostatic surgery, history of AUR within 3 months prior to study entry, 5-ARI use within 6 months (or dutasteride within 12 months) of entry or use of an α-blocker or phytotherapy for BPH within 2 weeks prior to entry.<br>The simulated baseline distribution of IPSS scores, prostate volume, maximum urinary flow and PSA values ensured patterns comparable to the observed covariate distributions in the pooled data from all studies (listed in Supplementary Table S1). Similarly, the simulated distribution of all other relevant clinical covariates identified as significant in the final model was based

	on the observed covariate distributions in the pooled population.
**Study visits**	Similar to the design of a typical clinical trial, IPSS was measured at study entry (baseline) and at 1, 2, 3, 4, 5, 6, 12, 18, 24, 30, 36, 42 and 48 months after start of treatment. Screening measurements were not included.
**Treatment arms**	Patients included in the CTS were assumed to be untreated at baseline. In addition, we assumed the assigned treatment was initiated with minor delays following LUTS/BPH diagnosis. Our seven-arm, virtual trial had one reference and six alternative treatment scenarios:<br>• Reference: daily dosing of 0.5 mg dutasteride plus 0.4 mg tamsulosin combination therapy started immediately after LUTS/BPH diagnosis;<br>• Scenario 1: tamsulosin monotherapy (0.4 mg) started immediately after LUTS/BPH diagnosis;<br>• Scenarios 2: tamsulosin monotherapy (0.4 mg) until month 1 after LUTS/BPH diagnosis; then switching to daily dosing of 0.5 mg dutasteride plus 0.4 mg tamsulosin combination therapy up to month 48.<br>• Scenario 3: tamsulosin monotherapy (0.4 mg) until month 3 after LUTS/BPH diagnosis; then switching to daily dosing of 0.5 mg dutasteride plus 0.4 mg tamsulosin combination therapy up to month 48.<br>• Scenario 4: tamsulosin monotherapy (0.4 mg) until month 6 after LUTS/BPH diagnosis; then switching to daily dosing of 0.5 mg dutasteride plus 0.4 mg tamsulosin combination therapy up to month 48.<br>• Scenario 5: tamsulosin monotherapy (0.4 mg) until month 12 after LUTS/BPH diagnosis; then switching to daily dosing of

	0.5 mg dutasteride plus 0.4 mg tamsulosin combination therapy up to month 48.<br>• Scenario 6: tamsulosin monotherapy (0.4 mg) until month 24 after LUTS/BPH diagnosis; then switching to daily dosing of 0.5 mg dutasteride plus 0.4 mg tamsulosin combination therapy up to month 48.
**Statistical methods:**	The predicted responder rate [defined as proportion of patients who showed CMI at month 48] was selected as the primary endpoint and analysed using a log-rank test. A *t*-test was applied to the difference in IPSS relative to baseline at different visits after start of treatment. For completeness, the proportion of subjects transitioning across different symptom-severity groups was calculated for each virtual treatment arm along with the absolute difference in IPSS at different visits up to month 48. The proposed simulated scenarios aimed at reaching statistical significance with high statistical power (>90%) and low type 1 error ($\alpha \leq 0.05$). Whilst usually no predefined effect size has been set for IPSS change from baseline, previous investigations have shown that 90% statistical power is achieved with a group size of 296 patients when assessing mean differences between treatment groups of 1.6, with a standard deviation of 6, and a 0.05 level of significance [15].
**Assumptions:**	• Interindividual variability in pharmacokinetics was assumed to have a minor impact on treatment response, as the currently approved dose levels of tamsulosin-dutasteride yield nearly maximum pharmacological effect. Variation in response was therefore assigned primarily to interindividual differences in disease-specific parameters.

	• All CTS scenarios were implemented under the assumption of perfect adherence to treatment. In real-life conditions, different adherence patterns may occur, depending on symptom severity and/or comorbidities, which may significantly alter the predicted differences across CTS scenarios.<br>• As drop-out in real clinical trials appears to be non-informative (i.e. at random), treatment scenarios were implemented without dropout.<br>• Parameter estimates obtained from the pooled patient database (N=10,236 had dosing records) were assumed to be sufficiently precise to replicate the performance of the treatment in a wider population, as observed in clinical practice. However, we recognise that inclusion/exclusion criteria may not fully reflect the LUTS/BPH population eligible for treatment with α-blockers and 5-ARI in clinical practice.

5-ARI, 5α-reductase inhibitor; AUR, acute urinary retention; CMI, clinically meaningful improvement; CTS, clinical trial simulations; IPSS, International Prostate Symptom Score; LUTS/BPH, lower urinary tract symptoms due to benign prostatic hyperplasia; PSA, prostate-specific antigen.

Table S3. Demographics and baseline characteristics of the patient population used for evaluation of the effect of immediate versus delayed start of tamsulosin-dutasteride combination therapy using CTS. The total number of patients at each visit includes those who transitioned to combination therapy. To allow direct comparison with a typical clinical trial, data shown correspond to results from a single trial replicate.

Baseline demographics	Combination therapy		Tamsulosin		Tamsulosin non-responders to combination therapy at 1 month		Tamsulosin non-responders to combination therapy at 3 months		Tamsulosin non-responders to combination therapy at 6 months		Tamsulosin non-responders to combination therapy at 12 months		Tamsulosin non-responders to combination therapy at 24 months	
	n	Mean (min – max)	n	Mean (min – max)	n	Mean (min – max)	n	Mean (min – max)	n	Mean (min – max)	n	Mean (min – max)	n	Mean (min – max)
**Age (years)**	500	66.9 (50–88)	500	66.4 (51–94)	500	65.9 (50–86)	500	65.8 (50–86)	500	66.7 (50–86)	500	65.9 (48–84)	500	66.0 (50–84)
**Height (cm)**	499	173.8 (152–195)	499	173.4 (144–202)	499	174.4 (153–193)	498	174.6 (152–198)	495	173.9 (143–203)	500	173.9 (132–194)	498	173.5 (149–198)
**BMI**	499	27.4 (12.4–59.5)	498	27.5 (18.5–57.5)	499	27.2 (16.7–41.0)	498	27.9 (16.9–59.7)	495	27.6 (17.3–57.8)	499	27.6 (18.1–56.5)	498	27.3 (15.9–50.0)
**Alcohol use (N/Y)**	197/300		197/303		197/301		192/305		194/305		184/312		193/306	
**Race (White/Black/ Hispanic/Asian) (n)**	452/6/14/22		454/8/12/19		446/8/17/26		455/9/12/18		460/14/8/11		459/12/9/15		457/6/19/14	

**Duration of LUTS/BPH (years)**	480	5.0 (0.1–30.0)	479	5.2 (0–40.0)	486	5.1 (0.2–34.8)	478	5.2 (0.1–34.0)	476	5.3 (0.04–30.0)	482	5.0 (0.1–54.8)	482	5.0 (0.02–38.0)
**Time from LUTS/BPH diagnosis (years)**	491	3.5 (-0.6–24)	494	3.7 (0–40.0)	495	3.4 (0–30.0)	491	3.7 (0–34.0)	491	3.6 (0–40.0)	495	3.3 (0–34.8)	489	3.5 (0–34.0)
**Baseline prostate volume (cm^3)**	433	53.1 (27.8–290.2)	453	53.7 (26.1–183.0)	455	55.4 (28.2–178.4)	442	54.8 (29.4–147.7)	449	54.6 (27.9–223.6)	443	56.9 (24.6–296.9)	448	53.9 (25.7–145.8)
**Baseline serum PSA (ng/mL)**	498	4.1 (1.5–12.8)	498	4.0 (0.7–10.5)	499	4.2 (1.5–23.2)	494	3.8 (1.4–10.4)	500	3.9 (1.0–11.6)	499	3.8 (1.5–10.2)	500	4.1 (1.5–11.8)
**Baseline IPSS**	500	17.1 (8–35)	500	17.4 (8–35)	500	17.1 (8–35)	500	17.3 (8–33)	500	16.9 (8–34)	500	17.0 (8–35)	500	17.1 (8–35)

CTS, clinical-trial simulations; IPSS, International Prostate Symptom Score; LUTS/BPH, lower urinary tract symptoms due to benign prostatic hyperplasia; PSA, prostate-specific antigen.

Table S4. CTS results for each treatment arm based on a parallel-study design (single trial replicate). Upper panel: Overview of the patient population and treatment allocation at each study visit, including details of those transitioning from tamsulosin to tamsulosin-dutasteride combination therapy due to non-response to tamsulosin. Lower panel: Primary endpoint, i.e., proportion of responders (response rate) along with IPSS values at 48 months in patients responding to treatment. The difference in the proportion of responders in each virtual treatment arm relative to the combination-therapy arm [$Responders_{CT}$ – $Responders_x$ (%)] summarises the impact of immediate combination therapy. Results refer to a single trial replicate including placebo effect only at the initial treatment phase. Placebo effect is a key component of the initial response and can last more than 6 months, as assessed by its half-life. No studies included a placebo treatment arm for >2 years, so it was not possible to establish whether inter-individual differences might allow for a longer placebo effect.

Treatment duration	Baseline IPSS†	Tamsulosin	Combination therapy	Transition to combination therapy	Cumulative number of patients transitioning to combination therapy
Start of		3000	500	0	0
1 month	16 (8, 35)	2511	500	489	489
3 months	16 (8, 33)	2083	989	428	917
6 months	16 (8, 34)	1817	1417	266	1183
12 months	14 (8, 35)	1649	1683	168	1351
24 months	15 (8, 33)	1494	1851	155	1506
36 months		1494	2006	0	1506
48 months		1494	2006	0	1506

Treatment arm	Baseline IPSS‡	Response rate (%)§	IPSS at 48 months	$Responders_{CT}$ – $Responders_x$(%)
Combination therapy (CT)	17.1 (8–35)	79.2	6.32	
Tamsulosin	17.4 (8–35)	65.6	9.20	
Tamsulosin non-responders switching to CT at 1 month	17.1 (8–35)	77.8	6.66	1.4
Tamsulosin non-responders switching to CT at 3 months	17.3 (8–33)	78	6.92	1.2
Tamsulosin non-responders switching to CT at 6 months	16.9 (8–34)	75.2	7.62	4*
Tamsulosin non-responders switching to CT at 12	17.0 (8–35)	70.6	7.61	8.6*
Tamsulosin non-responders switching to CT at 24	17.1 (8–35)	71	7.85	8.2*

†Baseline IPSS (range) in patients who transitioned to combination therapy; patients who do not achieve clinically meaningful improvement with tamsulosin at the predefined visit; ‡baseline IPSS (range) in each treatment arm; §percentage of responders (IPSS drop ≥25% relative to baseline, i.e. CMI) at 48 months; *log rank test: p<0.01. CT, combination therapy; IPSS, International Prostate Symptom Score.

Table S5. Impact of immediate versus delayed start of tamsulosin–Dutasteride combination therapy on the magnitude of response (single trial replicate), as assessed by the proportion of patients showing improvement in IPSS ≥ 35%, ≥ 50% and ≥ 75% relative to baseline at month 48.

	**Percentage of patients showing improvement at month 48**			
**IPSS improvement**	**≥25%**	**≥35%**	**≥50%**	**≥75%**
Combination therapy (CT)	79.2	72.4	60	32.2
Tamsulosin	65.6*	59.4*	46*	16.6*
Tamsulosin non-responders switching to CT at 1 month	77.8	70.8	56.6*	29.2*
Tamsulosin non-responders switching to CT at 3 months	78	70.4	58.6*	27.6*
Tamsulosin non-responders switching to CT at 6 months	75.2*	64.8*	53.6*	22.4*
Tamsulosin non-responders switching to CT at 12 months	70.6*	64.6*	51.2*	24.4*
Tamsulosin non-responders switching to CT at 24 months	71*	63.2*	52.2*	19.4*

*log rank test: p<0.01. CT, combination therapy; IPSS, International Prostate Symptom Score.
